# Junctional Ectopic Tachycardia

**Published:** 2010-07-20

**Authors:** Johnson Francis

**Affiliations:** 1 Malabar Institute of Medical Sciences, Calicut, Kerala, India; 2 KMCT Medical College, Calicut, Kerala, India

**Keywords:** Junctional ectopic tachycardia, narrow complex tachycardia, post operative tachycardia

Junctional ectopic tachycardia (JET) often occurs in the setting of surgery for congenital heart disease [1]. A congenital variety of JET not related to any surgery has also been described [2]. While post surgical JET has a mortality upto 14% [1], congenital JET has a mortality upto 34% [3]. A case of JET associated with myocarditis, has also been reported [4]. Electrocardiographic diagnosis of JET is made by the presence of AV dissociation in a narrow QRS complex tachycardia at a rate of 170 to 260 beats per minute and JET usually occurs within the first 24 to 48 hours of surgery [5]. Younger children tend to have faster and incessant JET [6]. JET in the fetus can be diagnosed based on AV dissociation noted in superior vena cava/ascending aorta Doppler flow recordings [7].

Mildh et al identified 51 patients with JET among a group of 1001 children undergoing open heart surgery over a 5 year period, an incidence of about 5% [1]. Rekawek J et al also found a similar incidence with 21 cases of JET among 402 children operated for congenital heart disease [8]. 8% incidence of JET among 336 cases were noted by Batra AS et al [9]. Andreasen JB et al reported an incidence of 10.2% of JET among 874 children who underewent corrective cardiac surgery  [10].

In case of post operative JET, longer cardiopulmonary bypass time, higher body temperature and higher levels of postoperative troponin T or creatine kinase and high inotropic requirement were associated with JET. These patients also needed longer ventilatory support and intensive care, compared to controls matched for the same type of surgery [1,9-11]. Age less than 1 month, history of cardiac failure and an Aristotle score more than 4 were also associated with JET [12].

Intravenous infusion of cold saline in addition to surface cooling, to achieve a core temperature of 32-34 ºC was evaluated in a pilot study for the management of post operative JET in 10 patients recently [13]. The median heart rate decreased from 187 beats per minute to 158 beats per minute. They could achieve AV synchrony in all patients, either by restoration of sinus rhythm or successful atrial pacing. Usually atrial pacing will not affect JET, as is demonstated in the case report by Arshi et al [14] in this issue of the journal. Occasionally, extracorporeal mechanical oxygenation is needed to support the circulation until the arrhythmia gets controlled [15].

Magnesium supplementation during cardiopulmonary bypass decreased the incidence of JET in a randomized, double blind controlled trial involving 99 children who underwent pediatric cardiac surgery  [12].

Amiodarone was useful as first line drug in a group of 40 pediatric cardiac surgical patients, though it was effective in only about half of them. While sinus rhythm was achieved only in 7 of them, a significant decrease in heart rate could be achived in another 11 patients. The lower rate permitted effective atrial or AV sequential pacing to achieve AV synchrony [16]. 13 of 15 patients with JET treated with amiodarone infusion by Plumpton K et al [17] responded within 12 hours, with a median response time of 4.5 hours. Amiodarone is also effective in non-post operative JET. Analysis of data from 22 institutions reporting 99 cases of JET and 5 cases of accelerated junction rhythm reported an efficacy of 60% [6]. Combined infusion of flecainide and amiodarone has been used in a refractory case of post operative JET [18]. Neonates with postoperative JET and no evidence of myocardial ischemia have been successfully treated with intravenous flecainide after failure of conventional therapies [19].

Dexmedetomidine may be useful in the treatment of perioperative JET, as noted in a preliminary observational report [20]. Intravenous nifekalant has been successfully used in terminating two episodes of JET following repair of tetralogy of Fallot [21]. Preoperative use of propranolol was associated with a lower incidence of JET (38% vs 21%) [22].

Radiofrequency ablation and cryoablation has a success rate of about 80% in the management of JET [Collins 2009], but may be reserved to cases not responding to other pharmacological and non-pharmacological measures. The site of earliest retrograde conduction during tachycardia can be targetted for ablation. Plotting out the entire His bundle system can be done prior to ablation and may be useful for preserving atrioventricular conduction  [23].
